# Poorly differentiated clusters (PDC) in colorectal cancer: what is and ought to be known

**DOI:** 10.1186/s13000-016-0481-7

**Published:** 2016-03-22

**Authors:** Luca Reggiani Bonetti, Valeria Barresi, Stefania Bettelli, Federica Domati, Cristian Palmiere

**Affiliations:** Department of Diagnostic Medicine and Public Health, University of Modena and Reggio Emilia – Section of Pathology, Via del Pozzo, 41124 Modena, Italy; Department of Pathology, University of Messina, Via Consolare Valeria, 98125 Messina, Italy; Department of Diagnostic Medicine and Public Health, University of Modena and Reggio Emilia – Section of Internal Medicine, Via del Pozzo, 41124 Modena, Italy; CURML, chemin de la Vulliette 4, 1000, Lausanne, 25 Switzerland

**Keywords:** Poorly differentiated clusters, Colorectal cancer, Tumor grading

## Abstract

**Background:**

The counting of poorly differentiated clusters of 5 or more cancer cells lacking a gland-like structure in a tumor mass has recently been identified among the histological features predictive of poor prognosis in colorectal cancer.

**Main body:**

Poorly differentiated clusters can easily be recognized in the histological sections of colorectal cancer routinely stained with haematoxylin and eosin. Despite some limitations related to specimen fragmentation, counting can also be assessed in endoscopic biopsies. Based on the number of poorly differentiated clusters that appear under a microscopic field of a ×20 objective lens (i.e., a microscopic field with a major axis of 1 mm), colorectal cancer can be graded into malignancies as follows: tumors with <5 clusters as grade 1, tumors with 5 to 9 clusters as grade 2, and tumors with ≥10 clusters as grade 3. High poorly differentiated cluster counts are significantly associated with peri-neural and lympho-vascular invasion, the presence of nodal metastases or micrometastases, as well as shorter overall and progression free survival to colorectal cancer.

**Conclusion:**

The morphological aspects and clinical relevance of poorly differentiated clusters counting in colorectal cancer are discussed in this review.

## Background

The incidence of colorectal cancer (CRC) has gradually increased over the decades in developed countries, and is now the third most-commonly diagnosed cancer [1–4].

In histopathological routine practice, tumor grade represents one of the most important predictive factors of colorectal cancer (CRC) aggressiveness [5]. To date, the most widely used system of defining CRC’s histological grade is based on the percentage of tumor glands forming the mass. However, this system suffers from significant inter-observer variability due to the lack of objective methods with which to assess the amount of glandular components [6].

More recently, a novel histological grading system has been highlighted as a histopathological prognosticator of CRC. This system considers poorly differentiated clusters (PDCs) composed of ≥5 cancer cells present at invasive front of the tumor that lack full glandular formation, [1, 4, 7–9].

## Main text

**Fig. 1:**
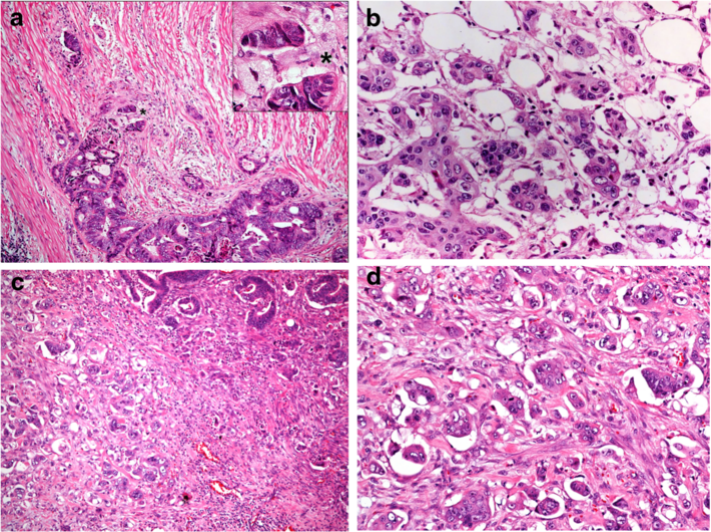
Grades of malignancy of CRC based on the highest PDC count. **a** PDC G1: <5 clusters [H&E stain 10×]. The particular of the clusters showing more than 5 undifferentiated cancer cells [asterisk - H&E stain 20×]. **b** PDC G2: 5–9 clusters [H&E stain 20×]. **c** PDC G3: ≥ 10 clusters at the fron of the tumor mass [H&E stain 10×]. **d** PDC G3: The same clusters at major resolution [H&E stain; 20×]

PDC can be evaluated in representative haematoxylin and eosin (H&E) stained slides that include the advancing edge of the tumor counted in the microscopic field under a ×20 objective lens (i.e. a 1 mm microscopic field). CRC can then be categorized into three grades of malignancy based on the highest PDC count: grade 1 (G1) count less than 5 (Fig. 1a), grade 2 (G2) range between 5 and 9 (Fig. 1b) and grade 3 (G3) 10 or more PDC clusters found (Fig. 1c).

Although PDC and tumor budding are similar in morphology given that neither have gland *silhouettes*, they represent two different entities. Indeed, by definition, tumor budding foci are smaller than PDC and made up of isolated cancer cells or small clusters of fewer than 5 elements [7, 10]. Thus, PDC are more easily recognizable at H&E stain and do not require the use of auxiliary immunohistochemical stains, such as cytokeratins. On the other hand, immunohistochemistry is mandatory for correct tumor budding identification, especially when it can be masqueraded by peritumoral desmoplastic tissue or inflammatory cell accumulation, as in leukocyte-rich peritumoral stroma [10–12].

The lack of information regarding PDC development has allowed for different hypothesis regarding their pathogenesis to be made. Their similar morphology suggests that PDC and tumor budding could represent sequential steps in tumor growth. This hypothesis is supported by the evidence of both PDC and tumor budding in the same tumor mass. This may serve as proof of possible sequential transformation of the latter into the former. Several studies in vitro have shown that tumor cells, singly or in large aggregates, can detach from the tumor mass and migrate into desmoplastic extracellular matrix with cohort-migration or through a mesenchymal-amoeboid transformation, activating an epithelial-mesenchymal transition process [13–19]. According to this development, PDC could represent the evolution of tumor buds or tumor podia, while they acquire proliferative and aggregative strength. Thus, PDC have been strongly associated with the up-regulation of Wnt/beta-catenin signaling pathways, such as metalloproteinase, disintegrin and L1-cell-adhesion-molecule (LICAM) [8, 20, 21] or beta-catenin [8, 20–22] as well as with the loss of pro-adhesion proteins such as caderin E [22, 23] or claudin [24]. These findings encourage the considerazion of PDC as directly involved in tumor dissemination and metastasis formation through direct invasion of lymph vascular channels. PDC could consequently assume a fundamental role in the definition of cancer aggressiveness and prediction of tumor behavior.

Recent studies have shown that PDC are strongly associated with vascular lymph invasion and lymph nodal metastases. Furthermore, they predict N+ status with higher sensitivity and specificity compared to other traditional, unfavorable histological factors of prognosis [25–27]. This data has been demonstrated in all TNM stages, including the early (pT1) CRC where PDC are correlated with tumor depth, particularly sub-mucosal invasion ≥ 1000 μn [27, 28].

These results encourage considering the presence and number of PDC as possible tools in risk assessment pertaining to nodal involvement. This is especially useful when conservative, local excision of low rectal carcinoma is used, when the lymph nodes cannot be examined or the number of examined lymph nodes is less than 12 (the minimum recommended for accurate staging in CRC) [27]. Furthermore, PDC grade has been reported as a significant predictor of occult micrometastases, defined as small metastatic deposits measuring less than 2 mm in a greater diameter detectable by immunohistochemistry or reverse transcription polymerase chain reaction in otherwise node negative (pN0) tumors [9, 26–29]. In particular, it has been demonstrated that PDC grade is significantly associated with the presence of lymph node micrometastases and may therefore be used in case selection cases for time-consuming, costly evaluation of occult nodal micrometastasis [27].

PDC’s efficacy in categorizing patients affected by CRC for prognosis has been evaluated in different studies. PDC grading has been demonstrated as a strong, independent prognostic factor in CRC [12, 26–29], where the high number of PDC (PDC G2 CRC and PDC G3 CRC) is strongly predictive of short, disease free survival and short disease specific survival, independent of pTNM stages [7, 28] and other unfavorable histological features, including traditional conventional WHO grading [7, 8, 26–29].

PDC-grading appeared more accurate in identifying stage I, subgroup with poor survival. Indeed, patients with stage I PDC-rich tumors have been shown to have an overall survival comparable to, or worse than that observed in subjects with stage III CRCs [9, 26, 27]. This data is relevant in oncological practice for this category of patients as well as in stage IIA subjects, in whom the post-surgical application of chemotherapy is controversial [25, 26].

Tumor grading based on PDC counting is applicable in more than 90 % of CRC endoscopic biopsies. It can reveal relevant information about the anatomical extent of the disease as well as biological proprieties of the tumor. When compared to traditional tumor grading, PDC-grading is more accurate in this context. In particular, high numbers of PDC in biopsy specimens are strongly predictive of unfavorable histological features, including infiltrating tumor borders, tumor budding, lymph vascular invasion, and perineural invasion in resection specimens, suggesting more aggressive behavior [25]. Similar to that observed in surgical specimens, a positive inter-observer agreement may be reached in the assessment of histological PDC grading in biopsy, which is higher than that achieved by using a conventional grading system [29]. These results may have remarkable clinical relevance in the choice of therapeutic management for patients affected by CRC, with particular regards to those with rectal cancer. Indeed, tumors with a low PDC grade could be submitted to local excision, whereas more invasive procedures, including anterior resection, might be reserved for those displaying a high number of PDC [30, 31].

Although PDC counting in biopsy has revealed a significant correlation with the corresponding surgical specimen that would suggest a lower number, there have been criticisms that the number of detectable clusters at biopsy may appear less since samples may not be taken from the deepest part of the tumor. Other problems encountered in PDC counting in biopsies are high tissue fragmentation, possible extensive necrosis, mainly in ulcerated tumors. Beyond these criticisms, thermal electro coagulation-induced cytological artifacts may reduce the quantity of evaluable cancer tissue and induce difficulties in PDC detection [25, 29]. The main difficulty is the distinction of PDC from transversally cut glandular crypts. These conditions increase confounding features and provoke discordance in PDC evaluation. However, bias can be avoided by excluding necrotic areas from the count and carefully considering all fragmented cluster cells detached from the sample using a critical approach.

A high PDC count has been significantly associated with CRC’s mutational status; in particular with KRAS mutation. The most frequent mutations have been observed in G12A and G12C in codon 12, and G13C and G13D in codon 13. These data give insight into the mechanisms of PDC development supposing that KRAS mutations might be responsible for their formation. The association of a KRAS mutation and high PDC grade, a higher depth colonic wall invasion and development of nodal metastases may account for a poorer prognosis of KRAS mutated CRC [32]. Although with no statistical significance, a similar trend has been viewed with V600E BRAF mutation.

## Conclusion

Due to its positive reproducibility and ease of histological detection, PDC are destined to assume more and more relevance in the histopathological description of CRC, as well as in clinical and oncological practice. Although no worldwide consensus has been reached thus far, PDC may foreseeable be introduced in a histological report, considered with other, commonly noted unfavorable histological features, to give further, important prognostic information for patient management.

### Ethical approval and consent to participate

Not applicable.

### Consent to publication

Not applicable.

### Availability of data and material

Not applicable.

## Abbreviations

CRC: Colorectal cancer
G1: Grade 1
G2: Grade 2
G3: Grade 3
H&E: Haematoxylin and eosin
LICAM: L1-cell-adhesion-molecule
PDC: Poorly differentiated cluster
TNM: Tumor, node, metastasis
